# Dual Inhibition of γ-Tubulin and Plk1 Induces Mitotic Cell Death

**DOI:** 10.3389/fphar.2020.620185

**Published:** 2021-01-29

**Authors:** Haruna Ebisu, Kana Shintani, Takumi Chinen, Yoko Nagumo, Shuya Shioda, Taisei Hatanaka, Akira Sakakura, Ichiro Hayakawa, Hideo Kigoshi, Takeo Usui

**Affiliations:** ^1^ Graduate School of Life and Environmental Sciences, University of Tsukuba, Tsukuba, Japan; ^2^ Department of Molecular Genetics, Division of Centrosome Biology, National Institute of Genetics, Mishima, Japan; ^3^ Department of Physiological Chemistry, Graduate School of Pharmaceutical Science, The University of Tokyo, Tokyo, Japan; ^4^ Faculty of Life and Environmental Sciences, University of Tsukuba, Tsukuba, Japan; ^5^ Graduate School of Pure and Applied Sciences, University of Tsukuba, Tsukuba, Japan; ^6^ Division of Applied Chemistry, Graduate School of Natural Science and Technology, Okayama University, Okayama, Japan; ^7^ Graduate School of Integrated Basic Sciences, Nihon University, Tokyo, Japan; ^8^ Microbiology Research Center for Sustainability (MiCS), University of Tsukuba, Tsukuba, Japan

**Keywords:** gatastatin, γ-tubulin, plk1, mitotic apoptosis, drug combination

## Abstract

α/β-Tubulin inhibitors that alter microtubule (MT) dynamics are commonly used in cancer therapy, however, these inhibitors also cause severe side effects such as peripheral neuropathy. γ-Tubulin is a possible target as antitumor drugs with low side effects, but the antitumor effect of γ-tubulin inhibitors has not been reported yet. In this study, we verified the antitumor activity of gatastatin, a γ-tubulin specific inhibitor. The cytotoxicity of gatastatin was relatively weak compared with that of the conventional MT inhibitors, paclitaxel and vinblastine. To improve the cytotoxicity, we screened the chemicals that improve the effects of gatastatin and found that BI 2536, a Plk1 inhibitor, greatly increases the cytotoxicity of gatastatin. Co-treatment with gatastatin and BI 2536 arrested cell cycle progression at mitosis with abnormal spindles. Moreover, mitotic cell death induced by the combined treatment was suppressed by the Mps1 inhibitor, reversine. These findings suggest that co-treatment with Plk1 and γ-tubulin inhibitors causes spindle assembly checkpoint-dependent mitotic cell death by impairing centrosome functions. These results raise the possibility of Plk1 and γ-tubulin inhibitor co-treatment as a novel cancer chemotherapy.

## Introduction

Microtubules (MTs) are dynamic polymers that are formed by the polymerization of α/β-tubulin heterodimers from MT nucleator γ-tubulin (Kollman et al., 2011). In mitosis, MTs form the essential scaffolding elements of the bipolar spindle that separates chromosomes with high precision. Errors in spindle function stimulate the spindle assembly checkpoint to block mitotic progression until all chromosomes are properly attached by MTs (Kops et al., 2005). Because a long-term blocking of bipolar spindle formation eventually leads to apoptosis, several α/β-tubulin inhibitors including taxanes and *vinca* alkaloids are used for cancer chemotherapy (Dumontet and Jordan, 2010). However, these inhibitors also cause severe side effects such as peripheral neuropathy. Furthermore, resistance mechanisms against α/β-tubulin agents such as expression pattern changes of tubulin isotypes and efflux pump systems have been reported. For these reasons, new antimitotic drugs with low side effects needed to be developed. Inhibitors against Eg5, CENPE, Aurora kinases and Polo-like kinase 1 (Plk1) are currently in clinical trials (Jackson et al., 2007; Casaluce et al., 2013).

Previously, we reported glaziovianin A (Chinen et al., 2013) derivatives gatastatin (Chinen et al., 2015) and gatastatin G2 (Shintani et al., 2020) as γ-tubulin specific inhibitors that induce short mitotic spindles with misaligned chromosomes without disrupting interphase MT networks. In addition to our observations, several studies have suggested that γ-tubulin may be a good candidate for the development of antitumour compounds with low side effects. First, γ-tubulin accumulates on the centrosome in prometaphase to facilitate bipolar spindle assembly (Khodjakov and Rieder 1999; Hutchins et al., 2010). Second, γ-tubulin is overexpressed in glioblastoma cells (Katsetos et al., 2007). Third, increased MT nucleation activity enhances the invasion activity of cultured cells (Godinho et al., 2014). Finally, centrosomal MT nucleation has been shown to be an attractive drug target (Yao et al., 2013). Thus, MT nucleation is an attractive target for new anticancer drug.

In this study, we evaluated the cytotoxicity of gatastatin and showed that co-treatment with Plk1 inhibitor BI 2536 exhibits strong toxicity. This combination induces mitotic cell death by activation of mitotic checkpoints and degradation of Mcl-1, an antiapoptotic protein. Therefore, our study raises the possibility that γ-tubulin and Plk1 are suitable drug targets for antitumor medicine development.

## Methods

### Cell Culture and Chemicals

HeLa cells were cultured in DMEM (Nacalai Tesque, Kyoto, Japan) supplemented with 10% FBS, 100 U/mL penicillin, 100 μg/ml streptomycin. HL60 and Jurkat cells were cultured in RPMI 1640 (Nacalai Tesque, Kyoto, Japan) supplemented with 10% FBS, 100 U/mL penicillin, 100 μg/ml streptomycin. Paclitaxel (Cat# 163-18614), vinblastine (Cat# 221-00751) and staurosporine (Cat#197-10251) were purchased from FujiFilm Wako Pure Chemical Corporation. BI 2536 was purchased from Funakoshi (Cat# A10134-5). Reversine was purchased from Sigma (Cat# R3904). RO-3306 was purchased from AdipoGen (Cat# AG-CR1-3515). Gatastatin was synthesized as previously described (Hayakawa et al., 2012). All chemicals were dissolved in dimethylsulfoxide (DMSO).

### Analysis of Cytotoxicity and Cell Cycle Progression

Cell viability was determined using the WST-8 assay. HeLa cells (3 × 10^3^ cells/well in 96 well plate) were treated with each compound (final DMSO concentration was 1.0%) for 48 h. 10 μL of the WST-8 assay reagents (Dojindo, Kumamoto, Japan) was added to the culture. After 1–4 h incubation, the absorbance at 450 nm was measured with an iMark microplate reader (BioRad), and the cell viability (control %) was determined. For the cell cycle progression analysis, HeLa cells (3 × 10^4^ cells/mL) were treated with the compounds (final DMSO concentration was 0.1%) for 24 h. After washing with phosphate-buffered saline (PBS), cells were fixed with 70% EtOH (−20°C). Fixed cells were subsequently stained with Muse Cell Cycle Reagent. DNA content were detected using a Muse Cell Analyzer (Luminex Corporation). Synergism was evaluated according to the Chou–Talalay CI method using CompuSyn software (CompuSyn, Inc.) (Chou, 2010).

### Fluorescent Microscopy

HeLa cells (3 × 10^4^ cells/mL) were treated with the compounds (final DMSO concentration 0.1%) for 24 h. Cells were fixed with cold MeOH for 5 min (−20°C). Cells were incubated with anti-pericentrin (1:2,000 dilution, Abcam, Cat# ab4448) and anti-α-tubulin (1:1,000 dilution, Santa Cruz, Cat# sc-32293) antibodies. After staining with Alexa^488^-conjugated anti-mouse IgG (1:2,000 dilution, Invitrogen, Cat#A11001) and Alexa^568^-conjugated anti-rabbit IgG (1:2,000 dilution, Invitrogen, Cat#A11011), cells were washed four times with PBS and mounted with ProLong Glass Antifade Mountant with NucBlue (Invitrogen, #P36981). The spindle MT and centrosome structures were observed under a Leica AF 6000 fluorescence microscope (Leica Microsystems, Wetzlar, Germany) equipped with a ×63 objective lens. Images of 40 sections at 0.25 μm intervals were collected. The position of the two pericentrin signals of mitotic cells were analyzed using ImageJ software and then their distance was calculated. Pericentrin signals on centrosomes were quantified with ImageJ using raw data with max projection from 40 z-stacks. For cell death analyses by time-lapse imaging, HeLa cells (3 × 10^4^ cells/mL) were seeded onto 35 mm glass-bottom dishes (Greiner-Bio-One, #627870). Before imaging, cells were treated with the respective compounds (final DMSO concentration was 1.1%). Time-lapse imaging of the cells was performed using a Confocal Scanner Box, the Cell Voyager CV1000 (Yokogawa Electric Corp.) equipped with a ×20 objective lens and the stage incubator for a 35 mm dish. Bright field images were taken every 15 min. Images were analyzed using the FIJI distribution of ImageJ.

### Immunoblotting

HeLa cells (3 × 10^4^ cells/mL) were treated with the compounds (final DMSO concentration 0.1%) for 24 h. Cells were washed once with PBS and lysed with lysis buffer. After sonication, the cells were placed on ice for 15 min. The cell extracts were boiled at 100°C for 3 min, separated by sodium dodecyl sulfate-polyacrylamide gel electrophoresis, transferred to a polyvinylidene fluoride microporous membrane (FujiFilmWako, #033-23813), and blocked with 5% skim milk (Megmilk Snowbrand, Sapporo, Japan). They were then probed with the appropriate primary antibody and HRP-conjugated anti-IgG secondary antibody, and detected by enhanced chemiluminescence (Nacalai Tesque, #02230-30). Images were visualized using Sayaka Imager (DRC, Tokyo). The band intensities of PARP, Mcl-1, BubR1, BubR1(phospho S670), and actin were measured with ImageJ and normalized by actin.

### Statistical Analysis

Statistical analysis of pole-to-pole distances and fluorescence intensities on centrosomes was performed with GraphPad Prism 6.1. A one-way ANOVA with Tukey’s multiple comparisons’ test was used to compare samples and to obtain adjusted *p*-values. The numbers of repeated experiments and sample sizes are indicated in the figure legends.

## Results

### Combination of Gatastatin and BI 2536 (gatastatin-BI 2536) Shows Synergic Cytotoxicity

**FIGURE 1:**
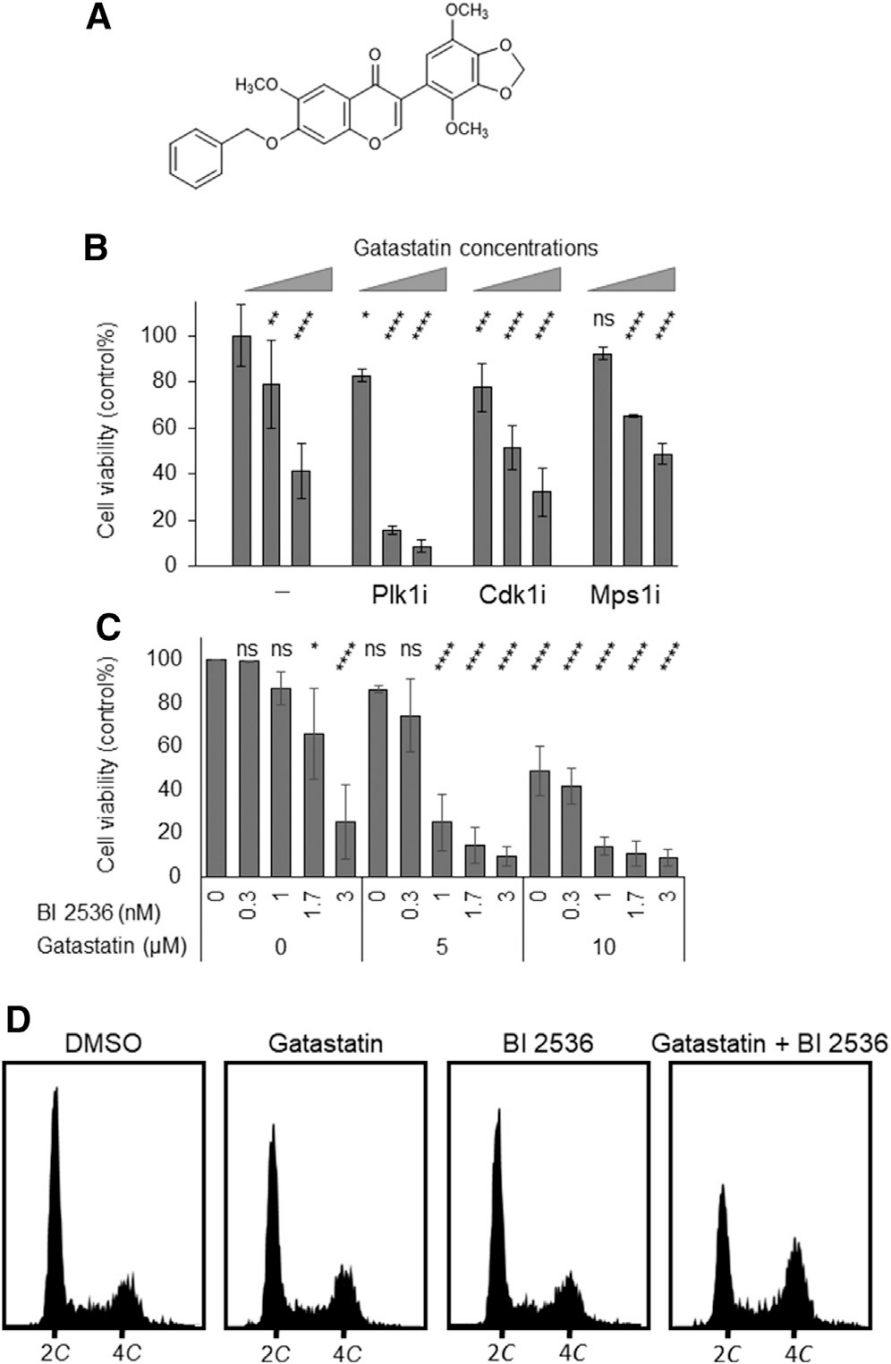
Co-treatment of gatastatin and Plk1 inhibitors shows improved cytotoxicity. **(A)** Chemical structure of gatastatin. **(B)** Plk1 inhibitor BI 2536, but not Cdk1 inhibitor RO-3306 and Mps1 inhibitor reversine, increased the toxicity of gatastatin. HeLa cells were treated with several drug combinations for 48 h and cell viability (DMSO control %) was determined with WST-8 assay. The concentrations of BI 2536, RO-3306 and reversine were 1 nM, 1 μM, and 0.3 µM, respectively. Gatastatin concentrations were 0, 5, and 10 µM. Error bars represent S.D. ANOVA was used to obtain *p* value. ns, no significance; *p < 0.05; **p < 0.01, ***p < 0.001; ****p < 0.0001. **(C)** Dose-dependent response of HeLa cells against gatastatin and BI 2536. HeLa cells were treated with each drug for 48 h and cell viability (DMSO control %) was determined with WST-8 assay. Error bars represent S.D. ANOVA was used to obtain *p* value. ns, no significance; *p < 0.05; **p < 0.01, ***p < 0.001; ****p < 0.0001. **(D)** Cell cycle profile of gatastatin- and/or BI 2536-treated cells. HeLa cells were treated with each drug for 24 h. After fixed with 70% EtOH, cells were stained with Muse Cell Cycle Reagent. DNA contents were analyzed with flow cytometer. The concentrations of BI 2536 and gatastatin were 1 nM and 5 μM, respectively.

To examine the antitumor activity of gatastatin, we investigated the cytotoxicity of gatastatin and other clinically used microtubule inhibitors, paclitaxel and vinblastine, against HeLa cells. The individual IC_50_ values of gatastatin, paclitaxel, and vinblastine were 9.02, 0.004, and 0.005 μM, respectively, indicating that gatastatin possesses only weak anti-proliferative activity. One method to increase the cytotoxicity of gatastatin is to combine it with other mitotic inhibitors. Therefore, we tested co-treatment with gatastatin and other mitotic kinase inhibitors, Cdk1 inhibitor RO-3306, Mps1 inhibitor reversine, and Plk1 inhibitor BI 2536. At the concentrations of each compound resulting in ∼80% cell viability, BI 2536, but not RO-3306 or reversine, drastically increased the cytotoxicity of gatastatin (Figure 1B). In paticular, 5 μM gatastatin and 1 nM BI 2536 (gatastatin-BI 2536) showed strong cytotoxicity (15% cell viability, Figure 1B). Because IC_50_ value of gatastatin and BI 2536 were 9.02 μM and 2.1 nM, respectively, these concentrations are about half of IC_50_ values of each compound. This effect was synergistic because 5 μM gatastatin and 1 nM BI 2536 showed only weak cytotoxicity on their own (86.2 ± 1.6% and 86.7 ± 7.7% cell viability, respectively; Figure 1C) and the combination index of combination treatment was 0.64. The synergic effects of gatastatin and BI 2536 were also observed in HL60 and Jurkat cell lines (Supplemental Figures 1A,B). Gatastatin-BI 2536 arrested cell cycle progression at the G2/M phase but the individual treatments did not when gatastatin or BI 2536 were used at that concentrations mentioned above (Figure 1D). The mitotic index of the combination-treated cells was 0.42 but that of the DMSO-treated cells was 0.08 (data not shown), indicating that the cell cycle arrest point in the combination-treated cells was mitosis. Because the same synergic effect was also observed in combination of gatastatin and HMN-214, another Plk1 inhibitor (Supplemental Figure 2), these results suggest that the Plk1 inhibitor increases the toxicity of a γ-tubulin inhibitor, and inhibits cell cycle progression in the M phase.

### Combination of Gatastatin and BI 2536 Inhibits Clustering of Pericentriolar Materials in Mitotic Cells

**FIGURE 2:**
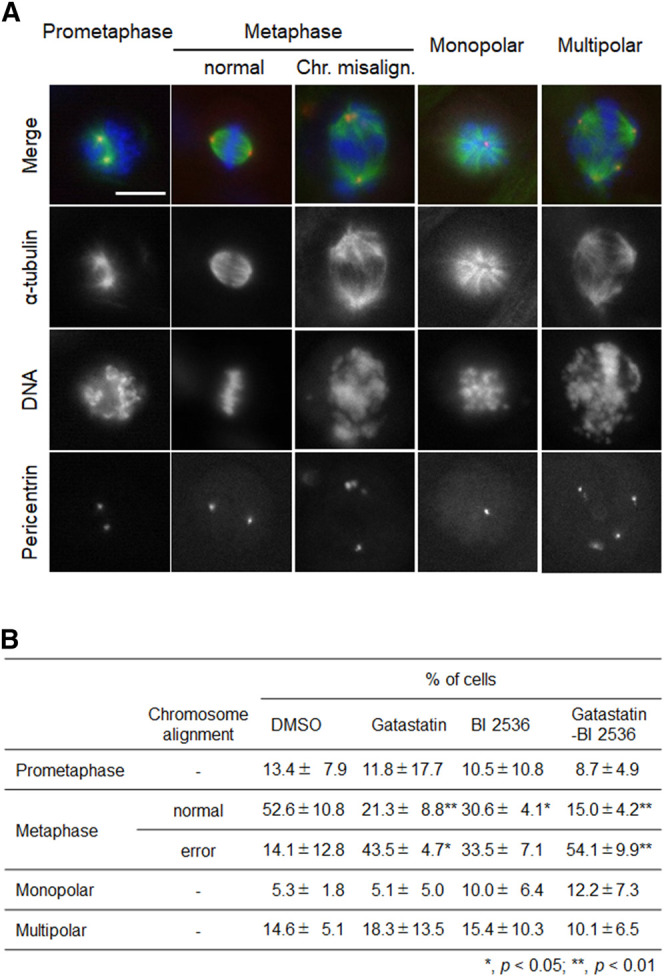
Combination treatment of gatastatin and BI-2536 induces spindles with chromosome misalignment. **(A)** Structure of mitotic spindle in the drug treated cells. HeLa cells were treated with the drugs for 24 h. Red, green and blue in the image represent pericentrin, α-tubulin and DNA, respectively. Scale bar, 10 µm. The concentrations of BI 2536 and gatastatin were 1 nM and 5 μM, respectively. **(B)** Classification of the morphology of the drug treated cells in **(A)**. Values represent average ±SD of three independent experiments. Greater than 56 cells of drug treated samples are examined for each experiment. *p < 0.05; **p < 0.01.

**FIGURE 3:**
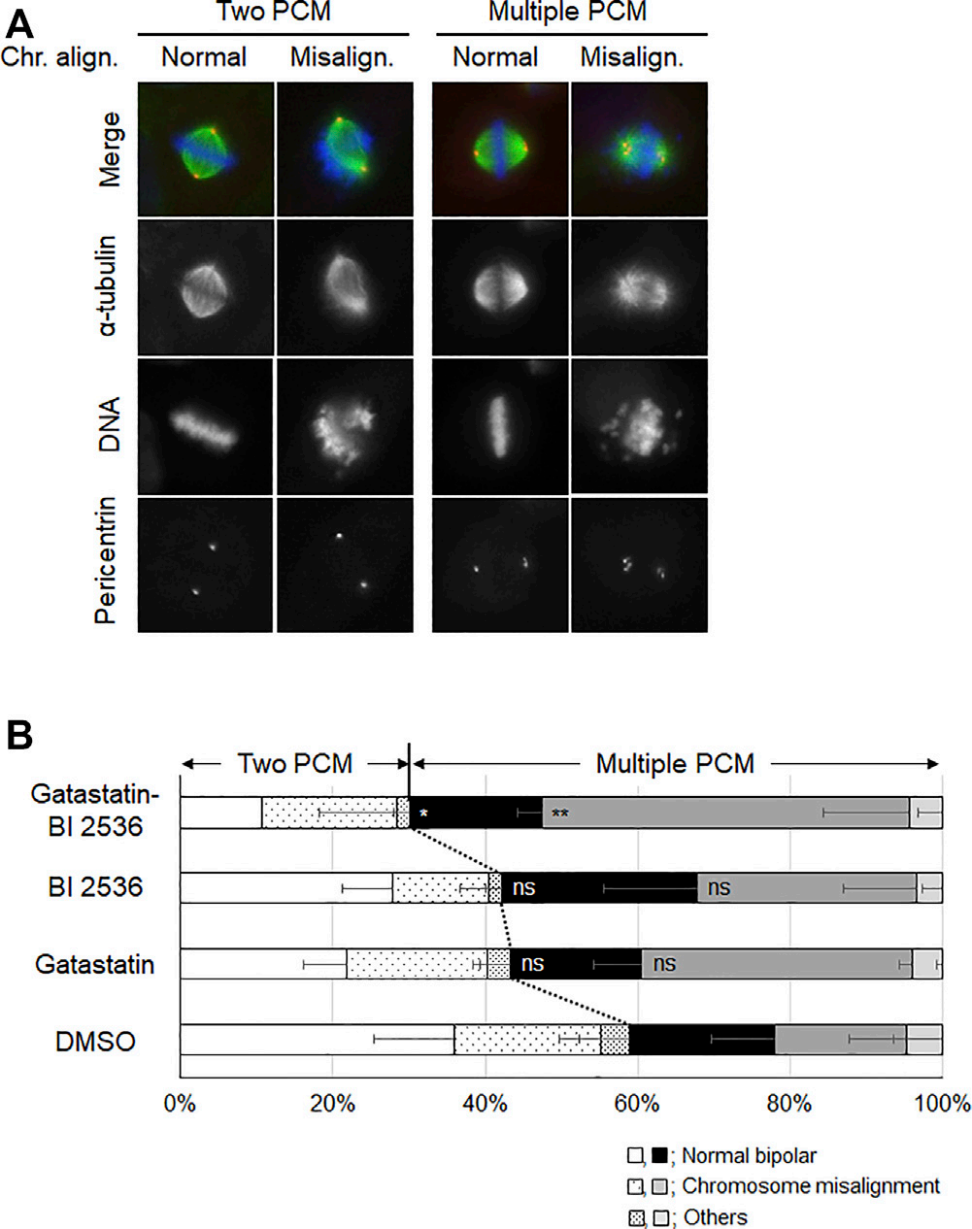
Combination treatment of gatastatin and BI-2536 impairs the proper PCM organization in mitotic cells. **(A)** Structure of mitotic spindle focusing on PCM organization and chromosome misalignment in the drug treated cells. HeLa cells were treated with the drugs for 24 h. Red, green and blue in the image represent pericentrin, α-tubulin and DNA, respectively. Scale bar, 10 µm. The concentrations of BI 2536 and gatastatin were 1 nM and 5 μM, respectively. **(B)** Classification of the morphology of the drug treated cells in **(A)**. Values represent average ±SD of three independent experiments. Greater than 44 cells of drug treated samples are examined for each experiment. Error bars represent S.D. ANOVA was used to obtain *p* value. n.s., no significance; *p < 0.05; **p < 0.01.

To investigate the mode of action of the synergic effect of gatastatin and BI 2536, we observed the spindle structure in the M phase (Figures 2A,B). Most spindles in DMSO-treated control cells showed normal bipolar spindles (52.6 ± 10.8%), and some bipolar spindles with chromosome misalignment were observed (14.1 ± 12.8%). In contrast, gatastatin, BI 2536, or gatastatin-BI 2536 treatment increased the amount of abnormal spindles, such as bipolar spindles with chromosome misalignment, and monopolar/multipolar spindles. Gatastatin significantly increased the quantity of bipolar spindles with chromosome misalignment (43.5 ± 4.7%), and this effect was enhanced by co-treatment with BI 2536 (54.1 ± 9.9%). Because it has been reported that Plk1 regulates the recruitment of γ-tubulin and pericentriolar materials on centrosomes (Haren et al., 2009), we quantified the pericentrin on spindle poles. However, we could not find any differences in pericentrin intensity at the spindle poles between DMSO-, BI 2536-, gatastatin-, or gatastatin-BI 2536-treated cells (Supplemental Figure 3A). We also found that there were no differences in the distances between the two centrosomes (pole-to-pole distance; Supplemental Figure 3B) or planar spindle orientations (Supplemental Figure 3C) at least at the concentrations we used. Instead, we noticed that gatastatin, BI 2536, or gatastatin-BI 2536 treatment increased the number of pericentrin signals, which was normally two signals per cell, indicating the fragmentation of pericentriolar materials (PCM) (Figure 3A). Within the bipolar spindle cells, the population containing fragmented PCM signals (multiple PCM in Figure 3B) increased in both gatastatin- and BI 2536-treated cells. Most of the fragmented PCM signals were observed in gatastatin-BI 2536 treated cells, suggesting that this effect was synergistic. Moreover, the number of bipolar spindles with chromosome misalignment significantly increased in gatastatin-treated cells, and this increase was further enhanced by co-treatment with BI 2536 (Figure 3B). These results suggest that the inhibition of Plk1 and γ-tubulin resulted in the impairment of proper PCM organization and induced abnormal spindle formation.

### Gatastatin-BI 2536 Induces Mitotic Cell Death

**FIGURE 4:**
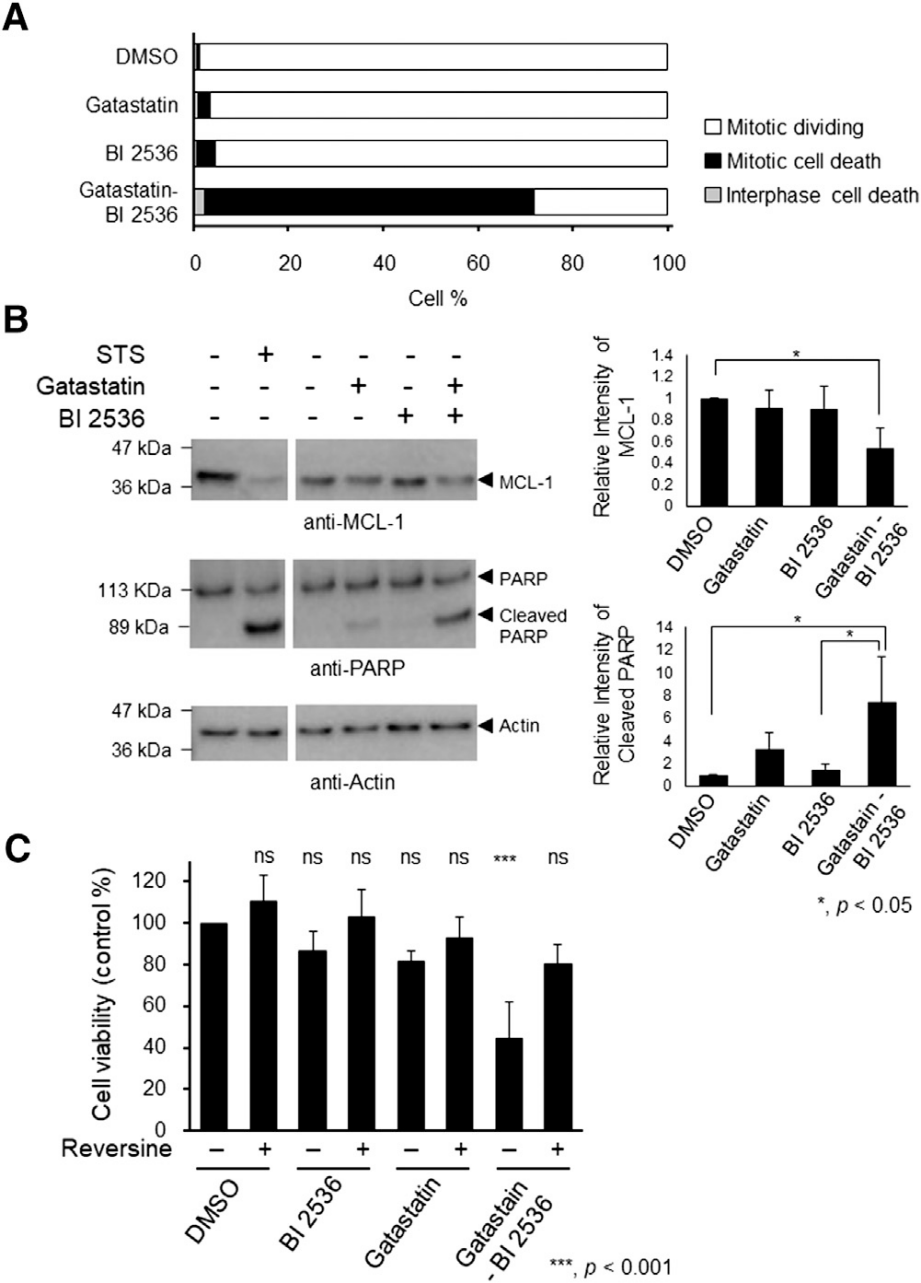
Combination treatment of gatastatin and BI 2536 induces mitotic cell death by degradation of Mcl-1 and activating mitotic spindle check points. **(A)** HeLa cells were treated with each drugs and time lapse observation was performed. Images were taken every 15 min for 24 h. The cell death in interphase and mitosis were counted and shown. The concentrations of BI 2536 and gatastatin were 1 nM and 10 μM, respectively. Greater than 463 cells from two independent experiments are examined. **(B)** Gatastatin-BI 2536 co-treatment induced decrease of Mcl-1 and cleavage of PARP. HeLa cells were treated with the drugs for 24 h and Mcl-1 decrease and PARP cleavage were analyzed by Western blotting. The concentrations of BI 2536 and gatastatin were 1 nM and 5 μM, respectively. Staurosporine (STS) was used as positive control (1 μM, 4 h treatment). Values represent average ±SD of three independent experiments. *p < 0.05. **(C)** Mitotic cell death induced by gatastatin-BI 2536 co-treatment was canceled by reversine. HeLa cells were treated with drug combinations for 48 h and cell viability (DMSO control %) was determined with WST-8 assay. The concentrations of BI 2536, gatastatin and reversine were 1 nM, 5 μM, and 50 nM, respectively. Values represent average ±SD of three independent experiments. ***p < 0.001; ns, no significance.

Mitotic inhibitors are known to kill cancer cells by inducing mitotic and post-mitotic cell death (Colin et al., 2015; Ohashi et al., 2015; Topham et al., 2015). To understand the mechanism of cell death induced by the combination of gatastatin and BI 2536, we performed time-lapse observation. Compared with the results in DMSO-treated cells, gatastatin- or BI 2536-treatment slightly increased cell death in mitosis, as indicated by cell death that occurred after the rounding up of cells (Figure 4A). On the contrary, gatastatin-BI 2536 co-treatment drastically increased mitotic cell death. Furthermore, gatastatin-BI 2536-treated cells, but not gatastatin- or BI 2536-treated cells, showed a decrease in antiapoptosis factor Mcl-1 and an increase in the cleavage of PARP, suggesting that this drug combination induces mitotic apoptosis (Figure 4B). It is known that mitotic cell death is induced by prolonged mitosis, which is caused by the activation of the spindle assembly checkpoint (SAC). Consistent with the observations in Figures 4A,B, phosphorylation of BubR1, a spindle checkpoint protein, was observed in gatastatin-BI 2536 co-treated cells (Supplemental Figure 4), and cell death caused by gatastatin-BI 2536 was completely averted by the Mps1 inhibitor reversine, which allows cells to progress into G1 phase by inhibiting the SAC (Figure 4C). Thus, gatastatin-BI 2536 triggered mitotic cell death by inducing mitotic checkpoint activation and decreasing Mcl-1 expression.

## Discussion

In this study, we revealed that the dual inhibition of γ-tubulin and Plk1 induces mitotic cell death by impairing mitotic spindle assembly. MT nucleation in mitotic spindles depends on centrosomes, chromatin, and the augmin complex (Prosser and Pelletier, 2017), and this process relies on γ-tubulin complex activity to nucleate MTs. Plk1 is an essential kinase for mitotic progression and regulates the recruitment of γ-tubulin and PCM on centrosomes (Haren et al., 2009; Johmura et al., 2011). However, there was no significant difference in the cell cycle progression and amount of pericentrin at spindle poles in BI 2536-treated cells compared with in DMSO-treated cells (Figure 1D, Supplemental Figures 3A,B, respectively). This suggests that BI 2536, at least at the concentrations used, did not have a strong impact on the recruitment of PCM or cell cycle progression. On the contrary, the occurrence of abnormal spindle morphology (bipolar spindles with chromosome misalignment and mono- or multipolar spindles) increased in BI 2536-treated cells (Figure 2B). The cells treated with gatastatin also had increased abnormal spindle morphology, but unlike with BI 2536, the number of bipolar spindles with misaligned chromosomes was substantial as we reported previously (Shintani et al., 2020). This phenotype was enhanced by combination treatment (Figure 2B). Furthermore, BI 2536 and gatastatin alone slightly increased the number of pericentrin signals, but gatastatin-BI 2536 greatly increased the number, which was normally two signals per cell (Figure 3B), suggesting that the inhibition of Plk1 and γ-tubulin induces PCM fragmentation. It is thought that PCM fragmentation causes spindle instability and activates SAC; therefore, both PCM fragmentation and chromosome misalignment probably contribute to SAC-dependent mitotic cell death in gatastatin-BI 2536 treatment (Figure 4). These results suggest that the inhibition of Plk1 and γ-tubulin results in abnormal spindle formation via the impairment of proper PCM organization and induces SAC-dependent mitotic cell death.

Several Plk1 inhibitors are currently in clinical trials (Jackson et al., 2007; Casaluce et al., 2013). Our study noted that the cytotoxicity of Plk1 inhibitors can be enhanced by a γ-tubulin inhibitor. Several reports suggest that α/β-tubulin inhibitors increase the toxicity of Plk1 inhibitors (Stehle et al., 2015; Weiß et al., 2015; Abbou et al., 2016; Czaplinski et al., 2016; Noack et al., 2018; Giordano et al., 2019). Therefore, the antitumor activity of Plk1 inhibitors is generally enhanced by both α/β-tubulin- and γ-tubulin-targeting drugs. Thus, Plk1 inhibitors may have potential for applications in combined treatment with α/β-tubulin and γ-tubulin inhibitors for cancer chemotherapy.

## Data Availability Statement

The original contributions presented in the study are included in the article/Supplementary Material, further inquiries can be directed to the corresponding authors.

## Author Contributions

HE, KS, TC, and YN: data collection, quantitative analysis, literature search, data interpretation, and graphic design. SS, TH, AS, IH, and HK: synthesis of gatastatin. TC and TU: conception and design, literature search, data interpretation, and manuscript preparation.

## Conflict of Interest

The authors declare that the research was conducted in the absence of any commercial or financial relationships that could be construed as a potential conflict of interest.
