# Supplementary material for: Dual Inhibition of γ-Tubulin and Plk1 Induces Mitotic Cell Death
Source: Front Pharmacol. 2021 Jan 29;11:620185. doi: 10.3389/fphar.2020.620185 (PMC7878676; doi:10.3389/fphar.2020.620185)
Supplement: Supplementary file 1 [file datasheet1.pdf]

Supplementary Data

A

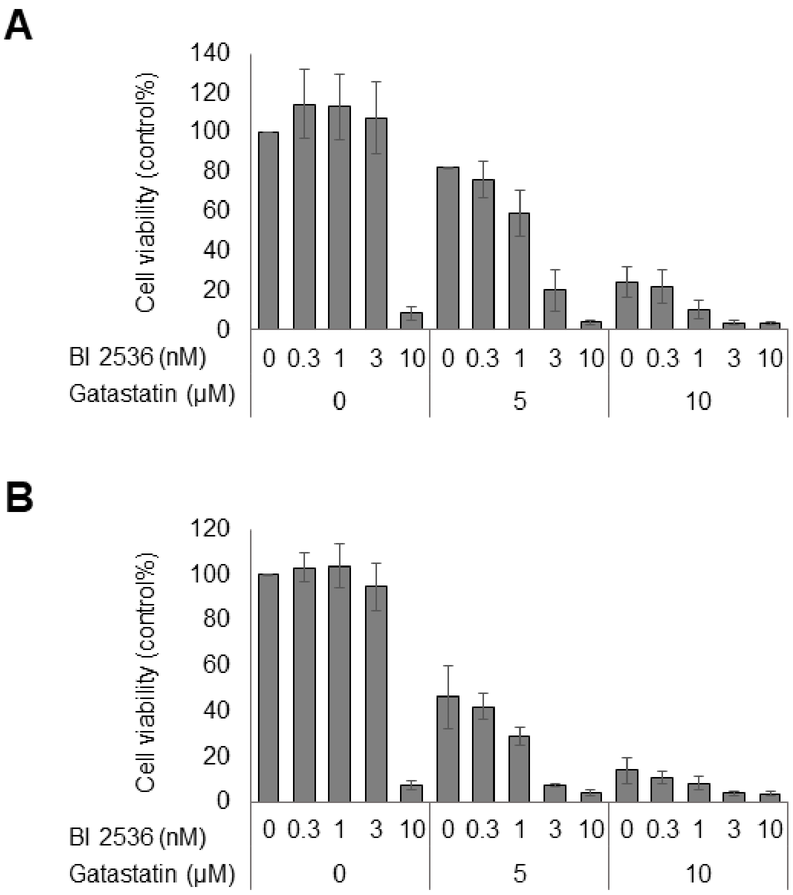

Supplementary Figure 1. Combination treatment of gatastatin and BI-2536 shows synergic cytotoxicity against HL60 cells (A) and Jurkat cells (B).

BI 2536 drastically increased the cytotoxicity of gatastatin in HL60 and Jurkat cells. The combination indexes were 0.80 and 0.68, respectively in co-treatment of 5 μM gatastatin and 3 nM BI 2536.

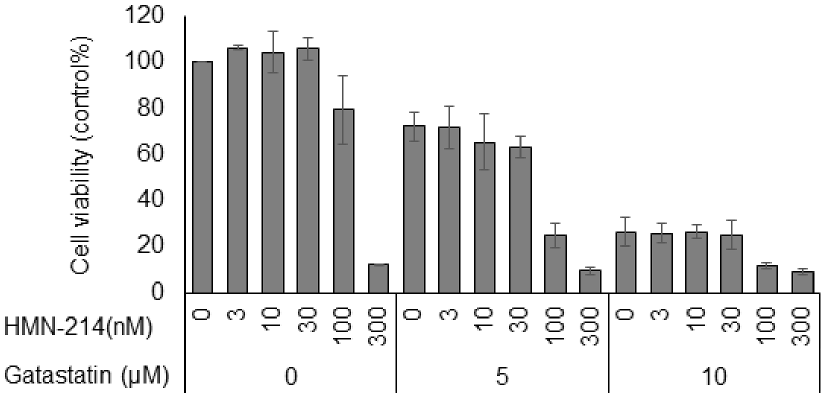

Supplementary Figure 2. Combination treatment of gatastatin and HMN-214 shows synergic cytotoxicity.

HMN-214 drastically increased the cytotoxicity of gatastatin in HeLa cell. The combination index was 0.75 in co-treatment of 5 μM gatastatin and 100 nM HMN-214.

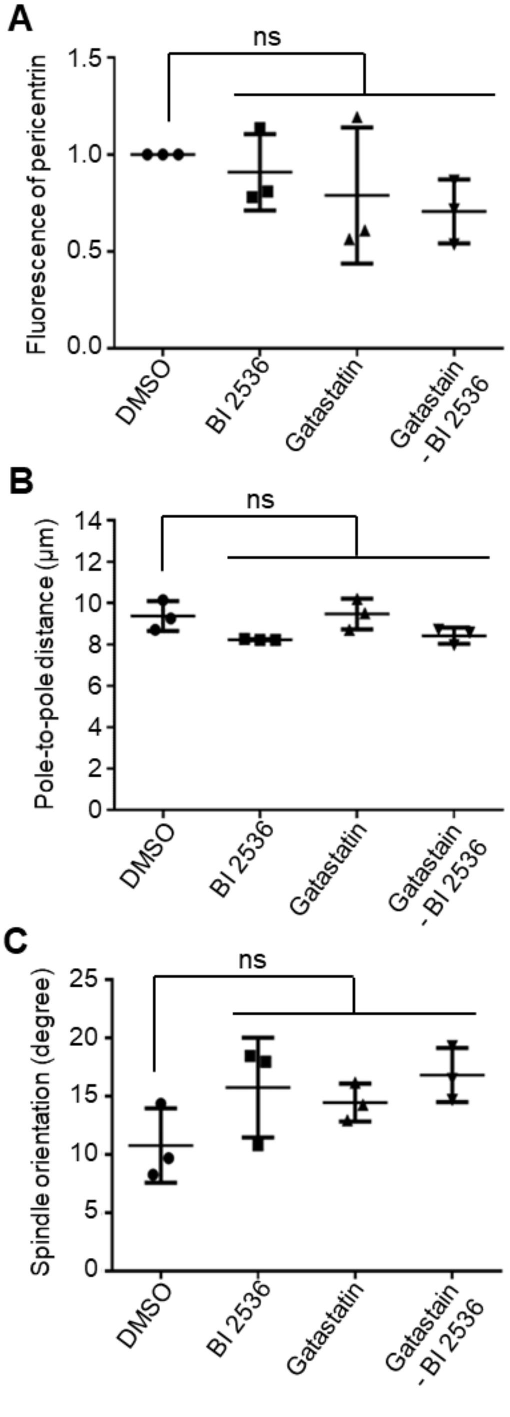

Supplementary Figure 3. Combination treatment of gatastatin and BI-2536 does not affect pericentrin intensity, pole-to-pole distance and orientations of bipolar spindles.

Pericentrin intensity at spindle poles **(A)**, Pole-to-pole distances **(B)**, and Planar spindle orientations **(C)** in DMSO-, BI 2536-, gatastatin-, and gatastatin-BI 2536-treated cells.

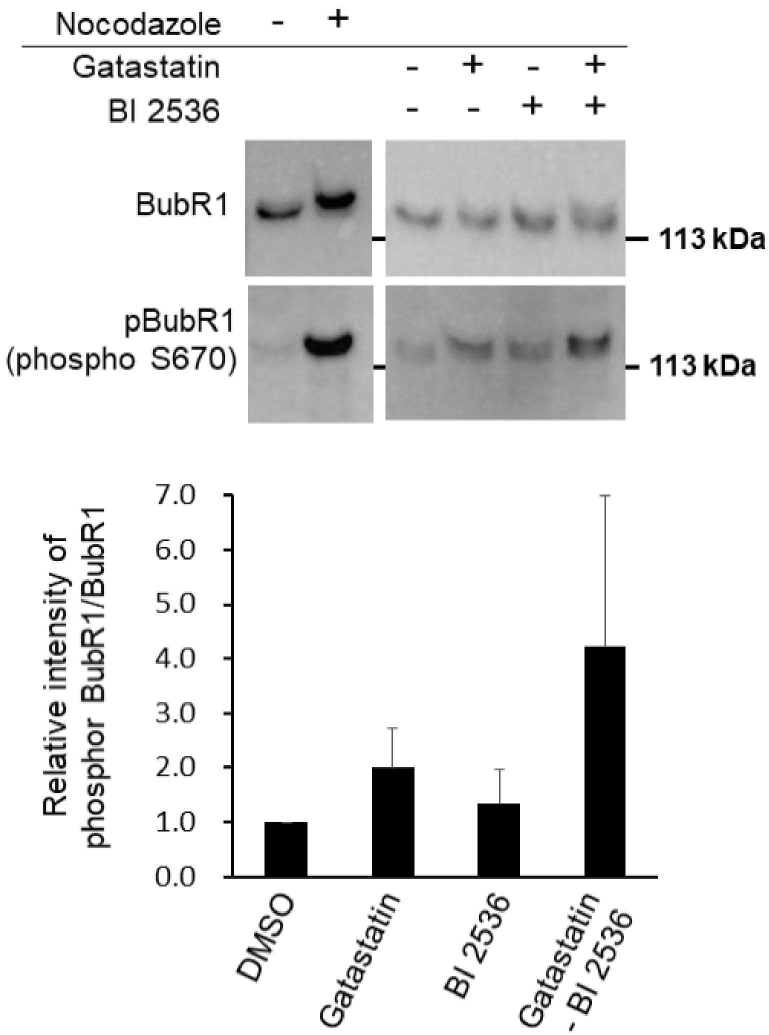

Supplementary Figure 4. Combination treatment of gatastatin and BI-2536 activates spindle assembly checkpoint (SAC).

SAC activation was judged by phosphorylation of BubR1. Nocodazole, a microtubule assembly inhibitor, was used positive control.
